# Breakfast and Snacks: Associations with Cognitive Failures, Minor Injuries, Accidents and Stress

**DOI:** 10.3390/nu3050515

**Published:** 2011-05-04

**Authors:** Katherine Chaplin, Andrew P. Smith

**Affiliations:** Centre for Occupational and Health Psychology, School of Psychology, Cardiff University, 63 Park Place, Cardiff CF10 3AS, UK; Email: chaplink1@cardiff.ac.uk

**Keywords:** breakfast, snacking, cognitive failures, accidents, injuries, stress

## Abstract

One strategy for examining effects of nutrients on cognitive function is to initially investigate foods that contain many different nutrients. If effects are demonstrated with these foods then further studies can address the role of specific nutrients. Breakfast foods (e.g., cereals, dairy products and fruit) provide many important nutrients and consumption of breakfast has been shown to be associated with beneficial effects on cognitive function. Isolating effects of specific constituents of breakfast has proved more difficult and it is still unclear what impact breakfast has on real-life performance. The present study provided initial information on associations between breakfast consumption and cognitive failures and accidents. A second aim was to examine associations between consumption of snacks which are often perceived as being unhealthy (chocolate, crisps and biscuits). A sample of over 800 nurses took part in the study. The results showed that frequency of breakfast consumption (varied breakfasts: 62% cereal) was associated with lower stress, fewer cognitive failures, injuries and accidents at work. In contrast, snacking on crisps, chocolate and biscuits was associated with higher stress, more cognitive failures and more injuries outside of work. Further research requires intervention studies to provide a clearer profile of causality and underlying mechanisms.

## 1. Introduction

The research reported in this article had two main aims. The first was to extend research on a well-established topic, the effects of breakfast on cognition, by examining associations between breakfast consumption and cognitive failures and accidents. A second aim was to investigate whether snacking also influenced these outcomes. The next section provides a brief overview of effects of breakfast.

Frequency of breakfast food consumption (e.g., cereal, dairy products, fruit and bread) is linked with a number of health benefits: better weight management; lower cholesterol; reduced risk of metabolic syndrome; better digestive functioning; fewer upper respiratory tract illnesses, and better mental health [1]. Regular breakfast consumption is associated with higher intake of key vitamins and mineral [2,3]. This may increase the likelihood of meeting nutritional requirements. Conversely, breakfast skippers may not make up for missed nutrients at other meals [4]. Breakfasts containing ready-to-eat-cereal may also improve the diet due to fortification with micronutrients and low fat levels. Indeed, a review of breakfast and the diet of adults confirms that breakfast eaters consume better quality diets that include more fiber and nutrients and fewer calories than the diets of breakfast skippers [5]. This has been confirmed in children and a review of 47 studies [2] showed that breakfast eaters have higher daily intakes of fiber, calcium, vitamin A, vitamin C, riboflavin, zinc and iron compared to breakfast skippers. The 2005*Dietary Guidelines for Americans* identify whole grains, fat-free and low fat milk and milk products, fruits and vegetables as “*foods to encourage*”. Popular breakfast foods help people meet recommendations for these food groups. Breakfast also contributes to whole grain intake (over 30% of the intake) which is known to reduce the risk of diabetes and coronary heart disease. Milk is the most commonly consumed breakfast food (consumed by over 50% of people who eat breakfast at home) and this, again, helps to meet dietary recommendations for this type of food. Similar results have been reported for fruit intake, with fruit or fruit juice consumption at breakfast being linked with greater total fruit intake over the day [6]. 

It is often thought that consumption of breakfast enhances performance, a suggestion which has arisen largely from a series of studies by Tuttle and colleagues over 40 years ago (“the Iowa Breakfast studies”). The main aim of these studies was to evaluate the effects of varying breakfast regimes on physiological performance but a number of the studies also included some tests of mental performance. In the first experiment of the series [7], they compared the effects of four breakfast regimes: (a) a heavy breakfast, (b) a light breakfast, (c) no breakfast and (d) coffee only. Results showed that in the no-breakfast condition, there was a tendency towards slower reaction times. However, this was the only condition in which caffeinated coffee was not given and the results may reflect this. This was replicated when the same subjects were re-tested. Five out of six of the females showed a significant increase in simple reaction time in the no-breakfast condition, while three out of six showed a significant increase in choice reaction time in the same condition. Clearly results from studies with such a small number of subjects must be treated with caution. 

They then carried out a similar experiment [8] comparing breakfast and no-breakfast conditions, with testing taking place three hours after breakfast. Six of the ten subjects showed no change in reaction time in the no-breakfast condition (as compared to breakfast), three showed a significant increase in reaction times, while one subject’s reaction time increased significantly during the no-breakfast condition. Again, it is difficult to draw confident conclusions from such a study. Another study [9] found no effect of breakfast on reaction times. Three breakfast conditions were compared: (a) bacon-egg and milk breakfast, (b) no breakfast, and (c) cereal and milk breakfast. Subjects (males aged 60 to 83 years) received the bacon-egg and milk breakfast for the first five weeks, followed by four weeks on no breakfast and four weeks on cereal and milk. Seven out of the eight subjects showed no change in reaction times during the course of the experiment. Although this experiment has the advantage that it examined the long term effects of breakfast, the small sample size, poor experimental design and the use of only a few measures of performance limits the value of the study. 

These early studies have been criticized for having small numbers of subjects, for producing inconsistent findings and for the use of subjective assessments [10]. The range of performance measures used was also small, being limited mainly to reaction time tasks. However, impaired performance associated with omitting breakfast was observed in other early studies with a variety of different types of breakfast. One study [11] assessed visual and motor functioning 2 h and 3 h after the consumption or omission of breakfast. The results showed that these functions were impaired when breakfast was not eaten compared to when it was. Another study [12] compared a standard breakfast with a no-breakfast condition. The volunteers were chosen so that half habitually ate breakfast and half no breakfast. A range of performance measures were employed: a visual search task, a short term memory task, vigilance task and a coding task. Testing was carried out in the late morning. Participants were tested on five occasions: once following their normal breakfast, twice following the standard breakfast and twice following no breakfast. A modified Latin-square design was used to balance order of conditions. The consumption or omission of breakfast did not alter performance. Rather, performance was most impaired when subjects changed from their normal meal. This led to the view that “the occasional omission of breakfast is more deleterious than the constant omission”.

Other research [13] has compared the effects of no breakfast and consumption of a high protein drink on spatial memory and immediate recall of a word list. Half the subjects were habitual breakfast eaters and half did not usually eat breakfast. Consumption of the high protein drink increased the speed with which both memory tasks were completed. Further research [14] has confirmed that breakfast improves aspects of memory and suggested that this may reflect several different mechanisms. Other studies have suggested that the size and composition of breakfast influence the post-meal response. One study [15] compared low fat/high carbohydrate, medium fat/medium carbohydrate, high fat/low carbohydrate and no-breakfast conditions. No clear differences in performance were observed as a function of type of breakfast but subjects given the low fat/high carbohydrate breakfast (which was most similar to their normal meal) reported improved mood compared to the other conditions. More recent research [16] has compared breakfasts that contained either high or low levels of carbohydrate, fat or protein. Better memory was found to be associated with consumption of meals that more slowly released glucose into the blood. This benefit of a low glycaemic index breakfast has been confirmed in animal studies [17] and in children [18,19].

The next section reports two studies [20,21] which examined the effects of breakfast on mood and a range of different aspects of performance. The type of breakfast was manipulated and the influence of caffeinated drinks examined. The experiments also investigated whether personality, eating habits, gender and previous night’s sleep modified any effect of breakfast on behavior. The first experiment examined the effects of two types of breakfast on sustained attention tasks (*i.e.*, tasks which show an effect of lunch), mood and cardiovascular functioning. Volunteers were given either caffeinated coffee or decaffeinated coffee after the meal (or no meal). This was done to investigate whether caffeine modified any effects of breakfast, and secondly, as a positive control to show that the tests used were sensitive to changes in state produced by caffeine [22]. 

In the first study a between subject design was used and volunteers were assigned to one of the six conditions formed by combining the three breakfast and two caffeine conditions. Volunteers were either assigned to a no-breakfast condition, a cooked breakfast condition or cereal/toast breakfast. After breakfast participants were either given de-caffeinated coffee or de-caffeinated coffee with 4 mg/kg body weight of caffeine tablets added to it. The results showed that breakfast had no effects on performance of sustained attention tasks. In contrast, caffeine improved performance of these tasks. No interactions between breakfast conditions and personality were found in any of the analyses. Similar results were found when gender was included as a factor. Smith *et al.* [21] examined effects of breakfast on performance of memory tasks. Consumption of breakfast improved recall and recognition of a list of words but had no beneficial effects on working memory or semantic memory tasks. Again, effects of breakfast were not modified by caffeine or by personality and gender. Breakfast had no effect on free recall in the late morning or after lunch, which suggests that the effects of breakfast on episodic memory are restricted to a few hours after the meal. 

Smith, Clark and Gallagher [23] extended the above results by showing that consumption of breakfast cereal may also improve spatial memory. However, the most robust effects of breakfast on memory are found in free recall tasks and these effects have been observed after consumption of high carbohydrate cereals and cereal bars [24,25]. Similarly, a mid-morning cereal bar may also have beneficial effects when consumed after a small breakfast [24].

There have been a few studies that have examined effects of breakfast in elderly adults. Early studies by Tuttle and colleagues found little evidence for an effect of breakfast on the cognitive function of the elderly. More recent studies have demonstrated both acute effects of breakfast and effects of breakfast habit. Kaplan *et al.* [26] found that carbohydrate intake was associated with improved performance of a short-term memory task whereas a protein breakfast was associated with reduced forgetting in a paragraph recall task. Smith [27] found that elderly adults, aged between 60 and 79 years, who ate breakfast cereal every day performed better on a test measuring intellectual functioning than those who consumed breakfast less frequently. It should be noted that this last result could reflect an effect of intelligence on breakfast consumption rather than a causal effect of breakfast consumption on intelligence. Further intervention studies are needed to assess the effects of breakfast on cognitive function in the elderly.

There have been a number of reviews of the effect of breakfast on the cognitive performance of adolescents and children [2,28,29] and the main findings can be summarised as follows. There have been over forty studies published on this topic in the last 60 years (see [29] for details of the literature). The results confirm the adult literature showing that breakfast has a beneficial effect on cognition, with the strongest support coming for improvements in memory. This effect is most readily apparent when nutritional status is compromised. Less is known about the effects of different types of breakfast and the role of breakfast size and composition requires further consideration. Wyon *et al.* [30] reported that children did better on tests of creativity, physical endurance and mathematical ability when they consumed a high energy breakfast than when they consumed a low energy breakfast. Michaud*et al.* [31] confirmed these results using a short-term memory task. Other studies [28] have shown that an oatmeal breakfast led to better performance (especially in girls) than ready-to-eat cereal. Most studies have investigated children rather than adolescents. A recent study of high school students [32] showed that breakfast had no effect on sustained attention but improved visuospatial memory in males. Studies of school breakfast programmes suggest that such interventions can have positive effects which may reflect an effect of the programmes on school attendance. 

Little is known about the real-life behavioral implications of consuming breakfast for adults. For example, a literature search revealed no information on breakfast and accidents and errors at work (or outside of work), road traffic accidents or driving performance, or on productivity at work. Recent research on caffeine has moved from the laboratory to epidemiological studies of consumption and human error and accidents. Smith [33] examined the impact of habitual caffeine consumption on performance and safety at work. The study involved secondary analyses of a database formed by combining the Bristol Stress and Health at Work and Cardiff Health and Safety at Work studies. In the first analyses associations between caffeine consumption and frequency of cognitive failures were examined in a sample of 1253 white-collar workers. The second set of analyses examined associations between caffeine consumption and accidents at work in a sample of 1555 workers who were especially at risk of having an accident. The results from the study demonstrated significant associations between caffeine consumption and fewer cognitive failures and accidents at work. After controlling for possible confounding factors it was found that higher caffeine consumption was associated with about half the risk of frequent/very frequent cognitive failures and a similar reduction in risk for accidents at work. Overall, the results confirmed that caffeine consumption may have benefits for performance and safety at work. 

Smith [34] conducted secondary analyses of a large epidemiological database to examine associations between caffeine consumption and cognitive failures (errors of memory, attention and action) in a non-working sample. Associations between caffeine consumption and physical and mental health problems were also examined. After controlling for possible confounding factors significant associations between caffeine consumption and fewer cognitive failures were observed. Overall, the results show that caffeine consumption may benefit cognitive functioning in a non-working population. This confirms earlier findings from working samples. This beneficial effect of caffeine was not associated with negative health consequences. This approach of examining associations with cognitive failures and accidents was used here to examine possible beneficial effects of consuming breakfast. When one is investigating a new topic it is sensible to also try and replicate established findings. Breakfast consumption has also been associated with lower levels of stress [35,36,37] and this was also examined here.

Recent research has examined the effects of snacking on well-being [38,39,40]. Little of this research has focused on cognitive functioning although there is evidence that “grazing” (eating a little but often) leads to better performance than consuming a smaller number of large meals [41]. In contrast to breakfast, where frequency of consumption rather than type of breakfast appears to be the most important factor, snacking frequency has less influence than type of snack. Research by Chaplin [42] has shown that snacks that are perceived as being unhealthy (chocolate, crisps, and biscuits-all perceived as unhealthy by over 80% of the sample) are associated with lower well-being scores. The second aim of the present research was to examine associations between this type of snacking and cognitive failures, accidents and stress.

## 2. Method

### 2.1. Participants

In total 870 people participated in the survey. The participants consisted of 790 females and 75 males. The mean age was 45 years (age range was 22-67 years). People were invited to participate in an advert placed in an issue 129 of the Royal College of Nursing (RCN) Bulletin. Letters were also sent to a random selection of 5000 people registered with the RCN and living in the South West of England. An information sheet was sent out with the questionnaires. This included a description about the aims of the project. Ethical approval was given by the Cardiff University, School of Psychology ethics committee.

### 2.2. Procedure

Letters were sent out with a blank address label. Participants were asked to write their address on the label and return it to the researchers in the freepost envelope provided. This label was used to post the questionnaire and no personal details were kept. People who responded to the advert in the RCN Bulletin were asked to phone and leave their address or Email with their address. The questionnaires were returned anonymously with no identifiers attached therefore no reminders or follow ups were completed. 

### 2.3. Materials

The questionnaire was designed to examine health and health-related behaviours, stress at work and outside work, and accidents, injuries and cognitive failures (both at work and outside work). Measures relevant to the present article are described below.

### 2.4. Breakfast Frequency

This was measured using an item from a standard food frequency questionnaire with ratings involving a 5 point scale from “Never” to “Everyday”. The questionnaire has been validated by comparisons with weighed dietary intake [43]. 

### 2.5. Frequency of Unhealthy Snacks

This measure was derived from a factor analysis of snacking behavior [42] and consisted of the sum of frequency of snacking of chocolate, crisps and biscuits (measured using a Likert scale from 0 (Never) to 6 (3 or 4 times a day).

### 2.6. Accidents, Injuries and Cognitive Failures

#### 2.6.1. At work

Cognitive failures at work were measured by the following single item:

“How frequently do you find that you have problems of memory (e.g., forgetting where you put things), attention (e.g., failures of concentration) or action (doing the wrong thing) at work?”

(responses made on a 5 point rating scale from “not at all” to “very frequently”)

This measure has been shown to be highly correlated with established measures of cognitive failure (e.g., the Cognitive Failures Questionnaire [44]).

The number of accidents at work requiring medical attention in the last 12 months were also recorded [45]. Similarly, the frequency of minor injuries at work was also recorded (responses made on a 5 point rating scale from “not at all” to “very frequently” [45]).

#### 2.6.2. Outside of work

Similar, questions were asked about the frequency of accidents, injuries and cognitive failures outside of work [45].

### 2.7. Stress at Work and Outside Work

Stress at work and stress outside of work were measured using single item 5 point scales from “Never” to “Extremely” [45].

### 2.8. Covariates

#### 2.8.1. The Work Environment

This section contained a number of standardized measures. Data from these questionnaires was collected as previous research has shown them to be strongly correlated with work outcomes [46]. The scores from these questionnaires were combined to form a negative job characteristics variable which was included as a covariate in all analyses involving work related outcomes (work stress; accidents at work; minor injuries at work and cognitive failures at work):

- Exposure to physical hazards and working hours [45];
- Demand-Control-Support [47];
- Effort-Reward Imbalance [48].

#### 2.8.2. Demographics

Items referring to age, gender, education, ethnicity and salary were included in this section. Age and gender were related to the outcomes and used as covariates.

#### 2.8.3. Negative Affectivity

Negative affectivity was measured using the neuroticism scale of the Eysenck Personality Inventory [49].

#### 2.8.4. Other Health-Related Behaviors

Smoking, alcohol consumption and sleep problems were assessed [45] and used as covariates.

#### 2.8.5. Statistical Analysis

Backward step binary logistic regression was used to analyze the data including covariates. Regression models were used in order to examine whether breakfast and unhealthy snacking exhibit any effects on the outcome measures when other health related behaviors and demographics are taken into consideration. Non-daily consumption of breakfast was compared to daily consumption of breakfast. Frequent snacking of chocolate, crisps and biscuits (>3 snacks per week) was compared to less frequent snacking (<3 snacks per week). A list of all the covariates included in the models is given in Table 1. Goodness of fit statistics were examined (Hosmer-Lemeshow, Cox & Snell and Nagelkerke) along with standardized residuals (Cooks, Leverance and DFBetas). Linear regression was also used to test for evidence of collinearity. Unless otherwise stated all of these values were normal and did not warrant any further exploration. 

**Table 1:** Covariates included in the regression models.

Variable	Description
Alcohol Consumption	Less than 21 units per week for men/14 units per week for women compared with greater than 21 units per week for men/14 units per week for women.
Smoking	Current cigarette smokers were compared to those who did not currently smoke cigarettes.
Difficulty sleeping	Those currently suffering from difficulties sleeping were compared to those having no difficulties sleeping.
Gender	Males and females were compared.
Age	Age was compared based on a median split (22-45 years compared to 46-67 years).
Neuroticism	Median split (score of 10 or less was compared to a score of more than 10).
Total negative job score	Median split (score of 17 or less was compared to a score of more than 17).

## 3. Results

### 3.1. Eating Habits

Over half of the participants (62%) reported eating 3 meals a day. Participants generally ate cereal for breakfast, a sandwich for lunch and either a small or large cooked evening meal. The majority of participants ate 1-2 snacks per day, with 78% of participants eating at least 1 snack per day. Therefore the participants were generally eating 4-5 times per day. 

### 3.2. Breakfast Consumption

Forty-two percent of the sample never consumed breakfast and this group was compared with those who consumed breakfast. Breakfast consumption was found to be significant in the final model (after controlling for confounders) for the following outcomes: accidents at work, accidents outside work, minor injuries at work, cognitive failures at work, and work stress (see Table 2). Daily breakfast consumption was associated with a reduced risk of an accident, minor injury or cognitive failure at work and lower work stress.

**Table 2:** Summary table of logistic regression results for breakfast frequency.

Outcome *N* = 859	Model χ^2 a^	Goodness of fit ^b^	Odds ratio	95% confidence intervals	*P* value
Accident at work	χ^2^(3) = 26.63	χ^2^(5) = 1.05	0.54	0.32-0.91	0.022
Minor injury at work	χ^2^(3) = 50.59	χ^2^(6) = 7.68	0.56	0.42-0.79	0.001
Cognitive failures at work	χ^2^(5) = 47.61	χ^2^(8) = 7.63	0.71	0.50-0.99	0.046
Work stress	χ^2^(6) = 102.96	χ^2^(8) = 5.87	0.63	0.45-0.90	0.010

^a^ *p* < 0.001; ^b^ *p* > 0.05.

### 3.3. Frequency of Snacking

Frequency of snacking had no effect on the outcome measures considered here. This confirms the view that snacking *per se* is less important than type of snack.

### 3.4. Perceived Unhealthy Snacking

Unhealthy snacking (defined as consuming chocolate, crisps or biscuits more than 3 times a week) was found to be significant in the final model for the following outcomes: accidents at work, minor injuries at work, minor injuries outside work, cognitive failures at work, cognitive failures outside work and life stress (see Table 3) gives the details relating to unhealthy snacking. Unhealthy snacking was associated with more accidents and minor injuries at work, more minor injuries and cognitive failures outside work, more concerns about health and more life stress.

**Table 3:** Summary table of logistic regression results for frequency of unhealthy snacking.

Outcome *N* = 825	Model χ^2^^a^	Goodness of fit ^b^	Odds ratio	95% confidence intervals	*P* value
Accident at work	χ^2^(3) = 22.23	χ^2^(6) = 5.55	1.78	1.02-3.11	0.042
Minor injury at work	χ^2^(3) = 60.08	χ^2^(6) = 3.78	2.06	1.49-2.85	0.000
Minor injury outside work	χ^2^(4) = 32.25	χ^2^(7) = 3.89	1.53	1.14-2.07	0.005
Cognitive failures outside work	χ^2^(4) = 45.53	χ^2^(8) = 15.22	1.52	1.08-2.13	0.016
Life stress	χ^2^(3) = 98.33	χ^2^(6) = 1.21	1.59	1.16-2.18	0.004

^a^ *p* < 0.001; ^b^ *p* > 0.05.

A significant association was seen between daily breakfast consumption and low unhealthy snacking χ^2^(1) = 17.62, *p* < 0.001. Both breakfast and unhealthy snacking were, therefore, included in the same regressions along with the full set of covariates. The same results were found as reported above except that breakfast was no longer significant for accidents outside work (see Table 4). Stress at work and stress outside work were then included in the regressions and the effects of breakfast and snacking on cognitive failures, injuries and accidents were unchanged showing that they were not due to effects on stress.

**Table 4:** Summary table of logistic regression results when breakfast frequency and frequency of unhealthy snacking were included in the model.

Outcome (*N* = 809)	Variable	Model χ^2 a^	Goodness of fit ^b^	Odds ratio	95% confidence limits	*P* value
Accident at work	Breakfast	χ^2^(3) = 23.18	χ^2^(5) = 2.19	0.45	0.26-0.78	0.005
Minor injury at work	Breakfast	χ^2^(5) = 73.37	χ^2^(8) = 3.77	0.66	0.47-0.92	0.015
	Unhealthy snacking			1.95	1.40-2.71	0.000
Minor injury outside work	Unhealthy snacking	χ^2^(4) = 31.99	χ^2^(8) = 4.01	1.54	1.14-2.09	0.005
Cognitive failures at work	Breakfast	χ^2^(4) = 46.25	χ^2^(7) = 9.16	0.68	0.48-0.96	0.026
Cognitive failures outside work	Unhealthy snacking	χ^2^(4) = 43.20	χ^2^(8) = 16.81	1.51	1.07-2.12	0.018
Work stress	Breakfast	χ^2^(6) = 101.46	χ^2^(8) = 5.72	0.56	0.39-0.81	0.002
	Unhealthy snacking			1.61	1.13-2.29	0.008
Stress in life in general	Unhealthy snacking	χ^2^(3) = 98.50	χ^2^(6) = 1.63	1.57	1.15-2.16	0.005

^a^ *p* < 0.001; ^b^ *p* > 0.05.

## 4. Discussion

Breakfast consumption and unhealthy snacking showed significant associations with stress, accidents, minor injuries and cognitive failures at work. The reduced stress levels reported by breakfast consumers and the higher levels associated with unhealthy snacking confirms previous findings. Previous research had identified smoking, alcohol consumption, sleep problems, age and gender to be associated with accidents and injuries. Dietary factors, particularly breakfast and unhealthy snacking, were still strongly associated with accidents, injuries and cognitive failures while controlling for these other variables. Regular breakfast consumers were half as likely to have an accident or minor injury at work as irregular breakfast consumers. This possibly reflects the less frequent cognitive failures at work reported by breakfast consumers. High consumption of unhealthy snacks was associated with twice the likelihood of having a minor injury at work and also outside work. Although it is not clear how breakfast and unhealthy snacking affect accidents, injuries and cognitive failures, it is a relationship which warrants further attention and investigation. Increasing breakfast consumption and reducing unhealthy snack consumption could be used as the basis of a simple and cost effective intervention for health and safety in the workplace. 

Only a few of the potential confounders were controlled for in the current study and more research is needed to explore the associations between breakfast and unhealthy snacking. It is not possible to draw any firm conclusions about the mechanisms by which breakfast and snacking may influence accidents and injuries. One possible explanation is that high fat meals have been found to increase fatigue and decrease alertness [50]. Unhealthy snacks are generally high in fat, while most breakfast cereals are low in fat. In addition breakfast cereal and toast have been found to be associated with increased alertness. Other factors which have been shown to be associated with accidents and injuries also need to be taken into consideration for example stress and fatigue levels. Cognitive failures are lapses in concentration and attention and may also be affected by fatigue and alertness. All of these results were found while controlling for demographic factors and health related behaviors. Gender, age, smoking, alcohol consumption and difficulty sleeping were included for all of the analyses. These results imply that the positive associations between breakfast and health outcomes are not simply a reflection of the positive effects of a healthy lifestyle. Unhealthy snacking was negatively associated with health and well-being in the current sample. It also appears that unhealthy snacking is not just an indicator of an unhealthy lifestyle *per se*. Due to the cross-sectional nature of the study it is not possible to make any conclusions about causation and directionality. However it is unlikely that having an accident influences dietary intake. Intervention studies are required to properly explore the relationships between breakfast frequency, snacking frequency and snacking type, and health and well-being. 

The current sample only considered working health professionals, predominantly nurses and therefore was homogenous. The vast majority of the individuals in this sample were white females who did not smoke and only consumed small to moderate amounts of alcohol. Therefore the conclusions drawn from the current study cannot be generalized to other groups. The associations between snacking type and health and well-being need to be replicated in a general public sample. In addition vulnerable groups, for example children and the elderly should be considered as they may receive the most benefit from any interventions. Breakfast and unhealthy snack food consumption exhibit strong associations with human error and well-being. This was still found to be the case when controlling for other lifestyle and demographic factors which were associated with health outcomes. The beneficial effects of breakfast were found for accidents, minor injuries and cognitive failures in the workplace. Increasing breakfast consumption may be the basis of an intervention programme to improve health. In contrast, unhealthy snacking was largely associated with non-work outcomes and changing these may influence cognition and well-being in such contexts.

## 5. Conclusions

The results from the present study show that consumption of breakfast is associated with fewer cognitive problems and accidents at work. In addition, the results confirm that breakfast consumption is associated with lower stress levels. Further research must now determine the mechanisms underlying these effects and the role of specific nutrients. In contrast, snacking on chocolate, crisps and biscuits was associated with more cognitive problems and injuries as well as higher stress levels. This is a relatively new research area and further research is needed to replicate these findings and to address the issue of underlying mechanisms.
